# Supplementary material for: Neural representation of words within phrases: Temporal evolution of color-adjectives and object-nouns during simple composition
Source: PLoS One. 2021 Mar 4;16(3):e0242754. doi: 10.1371/journal.pone.0242754 (PMC7932185; doi:10.1371/journal.pone.0242754)
Supplement: S1 Appendix — (DOCX) [file pone.0242754.s001.docx]

**S1 Appendix**

The results of the 2 x 2 ANOVAs described in the text are:

1. 2 x 2 ANOVA comparing isolation/isolation against phrase/phrase:
2. Main effect of **context** at *245-295ms* and *510-650ms*;
3. Main effect of **word-category** at *120-355ms*;
4. no **interaction** effect.
5. 2 x 2 ANOVA comparing list/list against phrase/phrase:
   1. main effect of **context** at *70-100ms* and *440-470ms*;
   2. main effect of **word-category** at *250-350ms* and *605-650ms*;
   3. **interaction** effect at *95-195ms*
6. 2 x 2 ANOVA comparing isolation/isolation against list/list:
7. main effect of **context** at *60-175ms* and *405-650ms*;
8. main effect of **word-category** at *290-360ms*;
9. **interaction** effect at 1*45-190ms*
